# 24-hour movement behaviour profiles and their transition in children aged 5.5 and 8 years – findings from a prospective cohort study

**DOI:** 10.1186/s12966-021-01210-y

**Published:** 2021-11-06

**Authors:** Natarajan Padmapriya, Bozhi Chen, Claire Marie Jie Lin Goh, Lynette Pei Chi Shek, Yap Seng Chong, Kok Hian Tan, Shiao-Yng Chan, Fabian Yap, Keith M. Godfrey, Yung Seng Lee, Johan G. Eriksson, Jonathan Y. Bernard, Falk Müller-Riemenschneider

**Affiliations:** 1 Department of Obstetrics & Gynaecology and Human Potential Translational Research Programme, Yong Loo Lin School of Medicine, National University of Singapore, 12 Science Drive 2, MD1 Tahir Foundation Building, Level 12, Singapore, 117549 Singapore; 2 Saw Swee Hock School of Public Health, National University of Singapore, Singapore, Singapore; 3 Singapore Institute for Clinical Sciences, Agency for Science, Technology and Research (A*STAR), Singapore, Singapore; 4 Department of Paediatrics, Yong Loo Lin School of Medicine, National University of Singapore, Singapore, Singapore; 5 Khoo Teck Puat-National University Children’s Medical Institute, National University Health System, Singapore, Singapore, Singapore; 6 KK Women’s and Children’s Hospital, Singapore, Singapore; 7 Duke-National University of Singapore, Singapore, Singapore; 8 Lee Kong Chian School of Medicine, Nanyang Technological University, Singapore, Singapore; 9 Medical Research Council Lifecourse Epidemiology Unit, University of Southampton, Southampton, UK; 10 NIHR Southampton Biomedical Research Centre, University of Southampton and University Hospital Southampton NHS Foundation Trust, Southampton, UK; 11 Department of General Practice and Primary Health Care, University of Helsinki and Folkhälsan Research Center, Helsinki, Finland; 12 Université de Paris, Centre for Research in Epidemiology and StatisticS (CRESS), Inserm, Inrae, F-75004 Paris, France; 13 Berlin Institute of Health, Charite University Medical Centre, Berlin, Germany

**Keywords:** Movement behaviour, Sleep, Inactivity, Sedentary behaviour, Physical activity, Children

## Abstract

**Background:**

Time spent in movement behaviours, including physical activity (PA), sedentary behaviour (SB) and sleep, across the 24-h day may have distinct health consequences. We aimed to describe 24-h movement behaviour (24 h-MB) profiles in children and how profile membership changed from age 5.5 to 8 years.

**Methods:**

Children in the Growing Up in Singapore Towards healthy Outcomes (GUSTO) cohort were asked to wear an accelerometer (ActiGraph-GT3X+) on their wrist for seven consecutive days at ages 5.5 and 8 years to measure 24 h-MB patterns. Time spent in night sleep, inactivity (proxy for SB), light PA, moderate PA (MPA), and vigorous PA (VPA) per day were calculated using the R-package GGIR 2.0. Using latent profile analyses (*n* = 442) we identified 24 h-MB profiles, which were given animal names to convey key characteristics. Latent transition analyses were used to describe the profile membership transition from ages 5.5 to 8 years. Associations with sex and ethnicity were examined.

**Results:**

We identified four profiles, “Rabbits” (very high-MPA/VPA, low-inactivity and average-night-sleep), “Chimpanzees” (high-MPA, low-inactivity and average-night-sleep), “Pandas” (low-PA, high-inactivity and high-night-sleep) and “Owls” (low-PA, high-inactivity and low-night-sleep), among children at both time points. At ages 5.5 and 8 years, the majority of children were classified into profiles of “Chimpanzees” (51 and 39%, respectively) and “Pandas” (24 and 37%). Half of the sample (49%), particularly “Rabbits”, remained in the same profile at ages 5.5 and 8 years: among children who changed profile the predominant transitions occurred from “Chimpanzees” (27%) and “Owls” (56%) profiles to “Pandas”. Sex, but not ethnicity, was associated with profile membership: compared to girls, boys were more likely to be in the “Rabbits” profile (adjusted OR [95% CI]: 3.6 [1.4, 9.7] and 4.5 [1.8, 10.9] at ages 5.5 and 8 years, respectively) and less likely to be in the “Pandas” profile (0.5 [0.3, 0.9] and 0.4 [0.2, 0.6]) at both ages.

**Conclusions:**

With increasing age about half the children stayed in the same of four 24 h-MB profiles, while the predominant transition for the remaining children was towards lower PA, higher inactivity and longer sleep duration. These findings can aid development and implementation of public health strategies to promote better health.

**Study registration:**

This study was registered on 4th August 2010 and is available online at ClinicalTrials.gov: NCT01174875.

**Supplementary Information:**

The online version contains supplementary material available at 10.1186/s12966-021-01210-y.

## Background

The prevalence of non-communicable diseases (NCDs), such as diabetes and cardiovascular disease, is one of the major public health challenges globally [1]. The World Health Organization (WHO) estimated that nearly two-thirds of premature deaths in adults are associated with lifestyle behaviours, including physical inactivity, and childhood conditions, such as overweight and obesity [2]. Evidence further suggests that lack of physical activity (PA), high sedentary behaviour (SB) and insufficient sleep are associated with adverse physical, mental, and social health indicators in children and adolescents [3–6]. Moreover, reviews of longitudinal studies highlight that PA, SB and sleep behaviours track from childhood to adulthood [7–9]. In recent years, there has been a paradigm shift from the isolated focus on the health impact of a single behaviour to the combination of these behaviours for maximum health benefits [3, 10]. Therefore, investigating combined movement behaviour profiles of PA, SB and sleep in childhood is important for the identification of existing behavioural patterns and to examine their effects on health and well-being.

A 24-h day comprises a sequence of movement behaviours distributed on a continuum ranging from no movement to high-intensity movement: sleep, SB, light-intensity PA (LPA), moderate-intensity PA (MPA) and vigorous-intensity PA (VPA) [11]. However, previous studies identified profiles/clusters of children mainly based on PA and SB or combinations of the crude adherence to guidelines of at least 60 min of moderate-to-vigorous PA (MVPA) and no more than 2-h of screen-based SB. Sleep was rarely included in previous studies and the clusters were predominantly based on non-movement behaviours, such as one or more types of diet intake [10, 12, 13]. Identifying profiles based on the time spent on the full continuum of movement behaviours is important to understand how children allocate their time in a day.

Technological and methodological developments of accelerometry now enables measuring movement behaviours continuously over 24 h and several nights/days [14]. Placement of accelerometers on the wrist, instead of the traditional location on the hip/thigh, has contributed to this progress, since it is associated with a greater compliance and is more comfortable to the participants [14]. The wrist placement, however, goes along with the inability to detect lower body posture which is an essential element of SB definitions: any waking behaviour characterized by energy expenditure ≤1.5 metabolic equivalent tasks (METs), while in a sitting, reclining or lying down posture [14, 15]. Instead, wrist-worn accelerometers measure inactivity during waking hours, which corresponds to energy expenditure ≤1.5 METs and can be viewed as a proxy for SB time [16].

Studies have reported that PA and sleep decrease, while SB increases with age [17–19]. These behavioural changes coincide with changes in children’s school curricular activities, in particular the transition from kindergarten (5–6 years of age) to primary school (7–8 years of age) [17]. However, such transitions remain poorly understood because the available evidence on movement behaviour profiles/clusters in children aged 12 years or below is largely based on cross-sectional studies [10, 12, 13]. Only one longitudinal study investigated how movement behaviour profile membership changes from age 6 to 9 years [20]. However, this study did not examine sleep and to our knowledge, profiles/clusters based on the combinations of the full continuum of movement behaviours using a 24-h time-use approach have not previously been investigated among children.

To address these gaps in the evidence in an Asian multi-ethnic cohort study, we investigated the movement behaviour profiles of children aged 5.5- and 8- years, and explored how the profile membership changed from age 5.5- to 8- years. Due to differences in values/norms and biological susceptibility among boys and girls, as well as differences in culture, beliefs and socio-economic status among different ethnic groups [21–23], movement behaviours may evolve differently in children of different sex and ethnicity [10, 20, 24–29]. We therefore further describe profile membership and transitions in profile membership from age 5.5 to 8 years according to sex and the three main ethnic groups (Chinese, Malay and Indian) residing in Singapore.

## Methods

### Study design and participants

We used data from the Growing Up in Singapore Towards healthy Outcomes (GUSTO) study, an ongoing multi-ethnic mother-offspring cohort study. The GUSTO study aims to investigate the role of early life factors on the child’s health and development. Between June 2009 and September 2010, pregnant women aged ≥18 years, of Chinese, Malay or Indian ethnicity with a same-ethnicity partner, Singapore citizens or permanent residents and attending their antenatal clinic visit at two major public maternity units (KK Women’s and Children’s Hospital, and National University Hospital) in Singapore were invited to participate. The protocol of the GUSTO study has been detailed previously [30]. The study received approval from ethics committees of the two study centres, the SingHealth Centralized Institutional Review Board and the National Healthcare Group Domain Specific Review Board in Singapore, and written informed consent was obtained from all participants. In total, 1450 women were recruited at their first trimester of pregnancy (< 14 weeks of gestation) and 18 women were recruited at delivery, and 1199 singleton babies were born and enrolled.

The children were followed up at the frequent clinic and home visits: at least four visits from birth to 1 year, at least two visits per year from 1 to 7 years and thereafter at least one visit per year in child’s birthday month [30]. Children’s date of birth, sex and ethnicity were extracted from medical records. Weight (to the nearest gram) and height (to the nearest 0.1 cm) of children were measured up to three times at age 5.5 years by trained research staff using a weighing scale (SECA model 803) and a stadiometer (SECA model 213, Hamburg, Germany), respectively, and repeated readings were averaged. BMI (in kg/m^2^) was derived from the average weight (in kg) divided by squared average height (in m^2^). Information about maternal age and educational level were obtained at recruitment as part of an interviewer-administered questionnaire, and household income was collected when the child was 5 years old as part of a self-administered questionnaire.

### Measurement of movement behaviours

ActiGraph GT3X+ (Actigraph Inc., Pensacola, FL), a triaxial accelerometer, was used to collect movement behaviour data on the children at age 5.5 and 8 years. During home (5.5 y) and clinic (8 y) visits, researchers attached an accelerometer with a non-removable strap on the child’s non-dominant wrist. Accelerometers were initialized to start recording at midnight after the visit, with a sampling rate of 80 Hz. Parents were asked to remove the device from the child’s wrist on the 9th day after the visit so that 7 complete days of continuous, 24-h data got captured. Data were downloaded in raw format (GT3X) and converted into raw, non-aggregated, comma-separated values file (CSV) format using the ActiLife software (version 6.13). Raw data were then processed in R software using the GGIR package (version 2.0) [31, 32].

Accelerometer devices are calibrated relative to gravity thus the raw acceleration was expressed in gravity (*g* units; 1 *g* = 9.81 m.s − ^2^). The vector magnitude was taken from the three axes raw signals and then subtracted by one gravity (*g*) after that negative values were rounded up to zero; this method is referred as Euclidian Norm Minus One (ENMO) in the literature. The resulting value was expressed in milligravity (m*g*, 1 m*g* = 0.00981 m.s − ^2^) [31]. Indices of 24-h activity were then calculated/aggregated based on 5-s epoch periods, since it is established that children largely engage in short bursts of movement [33]. Non-wear time was calculated based on the standard deviation (< 3 m*g*) and value range (< 50 m*g* in two of three-axis) of accelerometer axis, using the acceleration windows of 60 min with 15-min increments [31, 32, 34]. Days with ≥16 h/d of activity recordings (from midnight to midnight) were considered as valid, and children with at least two valid weekdays and one valid weekend day were included in the analysis.

Night sleep duration was calculated using GGIR default algorithm, as described by Van Hees et al. [35, 36]. Briefly, the Heuristic algorithm looking at Distribution of Change in Z-Angle (HDCZA) was applied to detect sustained inactivity bouts where the z-angle did not change by more than 5 degrees for at least 5 min, and then to determine the sleep window. Non-sleep time was classified based on ENMO cut-points as inactivity (ENMO < 35 m*g*), LPA (35 to 200 m*g*), MPA (200 to 707 m*g*) and VPA (≥707 m*g*) using prediction equations provided by Hildebrand et al. [16, 37]. Intuitively, inactivity time can be viewed as a proxy for SB time. However, it was not possible to determine posture and distinguish other types of inactivity from SB using wrist-worn accelerometers [14, 15]. Hence, in this study, we used the term inactivity as a proxy for SB. The weighted averages of time spent on each movement behaviour across all valid days, where weekend days are weighted 2/5 relative to the contribution of weekdays, were calculated and used in the current study. The weighted averages of MPA and VPA time per day were summed up to derive the MVPA time per day at both time points. MVPA (≥60 min/d) and sleep (9–11 h/d) variables were categorized based on meeting recommended (WHO/Canadian) guidelines [38, 39].

### Statistical analyses

Frequencies and percentages for categorical variables, means and standard deviations for continuous variables were calculated. Chi-square tests were performed to test differences between included and excluded children. Frequency distribution of PA, inactivity and sleep variables were visually inspected for normality, and outliers were excluded from the analyses (*n* = 1). These analyses were performed with STATA version 15.1 (StataCorp, College Station, Texas, USA, 2017). Latent profile analysis was conducted to derive a categorical latent variable that represents unobserved hidden subgroups (profiles) in the movement behaviour pattern of children aged 5.5 and 8 years using time spent in LPA, MPA, VPA, inactivity and sleep (continuous variables in min/d). Latent profile analysis uses a finite mixture modelling approach that accounts for time-use balance within 24-h day and provides data-driven categorization. The identified profiles can be defined by means and variances [40, 41]. We estimated how the profile membership changed from age 5.5 to 8 years using latent transition analysis, which is an extension of latent profile analysis [42, 43]. We conducted latent profile and latent transition analysis using a three-step approach, and then assessed the associations of sex and ethnicity with profile membership in Mplus (version 8.4, Muthen & Muthen) [44–46].

In the first step, we examined the 24-h movement behaviour profiles for children at both ages. We fitted cross-sectional latent profiles at age 5.5 and 8 years in separate models. We explored models with 2 to 7 profiles without any constrains or covariates. The final latent profiles model were identified on the basis of the best fit model, the number of profiles, their prevalence and distinguished 24-h movement behaviour pattern. Then, the corresponding longitudinal latent profile analysis models were examined using conditional models, where the number of profiles (configural similarity), mean and variance of 24-h movement behaviour indicators within each profile (structural and dispersion similarity) were similar across the time points as suggested by Morin and colleagues [47, 48]; this ensures interpretation of each profile is the same across the time points while maintaining the differences between profiles. The models were then compared for the goodness of fit using log-likelihood, Akaike’s Information Criterion (AIC), Bayesian Information Criterion (BIC) and sample-size adjusted BIC (SABIC) (for all models), where the lower value indicated a better fit [49, 50], bootstrapped likelihood ratio test (BLRT) for statistical significance (only for cross-sectional models), and higher posterior probability and entropy (≥0.75) [40, 49, 50]. Indices of model fit for latent profile analyses containing 2 to 7 profiles showed that AIC, BIC, SABIC and the log-likelihood decreased as the number of profiles increased in the models. The four-profiles model with similar mean and variance of movement behaviours of each profile across the time points was identified as the most parsimonious model in terms of goodness of fit, entropy and conceptually meaningful heterogeneous profiles at each time point compared other models (Supplementary Table 1). The identified profiles were given animal names in an attempt to convey key characteristics.

In the second step, we obtained the final latent variables and classification errors for each time point by fixing the measurement parameters, including the number of profiles, mean and variance, obtained from the final model in step one [44, 45, 51]. This step was repeated in the maximum sample at each time point to explore the consistency of profile membership. In the third step, we conducted latent transition analysis, the probability of transition between profiles across the time points was obtained after accounting for classification error derived in step two for each time point. This three-step method maintains the stability of profile membership within each time point during latent transition analysis [44, 45, 47, 51].

Finally, we examined the associations of sex and ethnicity with profile membership with the Wald test by using the Bolck-Croon-Hagenarrs (BCH) method. The BCH method is robust to measurement error of latent profiles and estimates the associations without influencing individual profile membership status [51, 52]. Additionally, sex- and ethnicity-specific transition probabilities of profile membership were derived using stratified latent transition analysis.

## Results

**Fig. 1:**
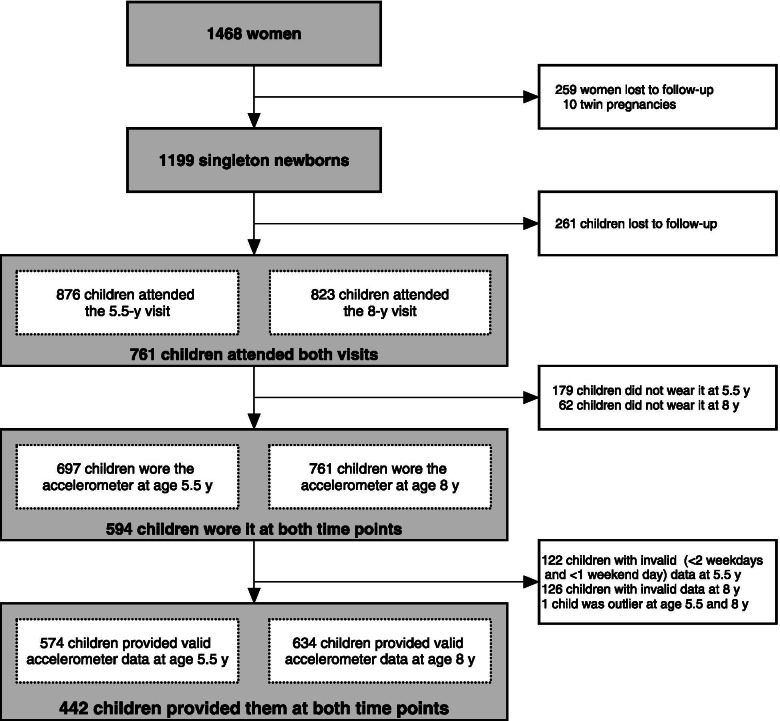
Flowchart of the participants of the present study

A total of 1199 children were enrolled in the GUSTO cohort. Of those, 574 and 634 children provided valid accelerometer measurements at ages 5.5 and 8 years, respectively (Fig. 1). Table 1 shows the characteristics of children at age 5.5 and 8 years. Among them, 442 children provided valid data at both time points and were included in the main analyses; included children were similar to excluded children with regard to sex and ethnicity.

**Table 1:** Characteristics of the children assessed by accelerometry at ages 5.5 and 8 years in the GUSTO cohort study

	Maximum sample		Overlapping sample	
	5.5 years (n = 574)	8 years (n = 634)	5.5 years (n = 442)	8 years (n = 442)
	% (n) or mean ± SD	% (n) or mean ± SD	% (n) or mean ± SD	% (n) or mean ± SD
**Child sex**				
Girls	47.7 (274)	48.4 (307)	47.5 (210)	47.5 (210)
Boys	52.3 (300)	51.6 (327)	52.5 (232)	52.5 (232)
**Ethnicity**				
Chinese	58.0 (333)	58.4 (370)	57.7 (255)	57.7 (255)
Malay	23.9 (137)	25.9 (164)	26.0 (115)	26.0 (115)
Indian	18.1 (104)	15.8 (100)	16.3 (72)	16.3 (72)
**BMI at age 5 years**				
Below median (< 15.0 kg/m^2^)	47.2 (271)	46.4 (294)	46.4 (205)	46.4 (205)
Median and above (≥15.0 kg/m^2^)	47.9 (275)	48.1 (305)	50.0 (221)	50.0 (221)
Missing data	4.9 (28)	5.5 (35)	3.6 (16)	3.6 (16)
**Maternal age at recruitment**				
< 27 years	23.3 (134)	22.4 (142)	21.7 (96)	21.7 (96)
27–33 years	39.9 (229)	40.4 (256)	40.5 (179)	40.5 (179)
> 33 years	35.2 (202)	35.5 (225)	36.2 (160)	36.2 (160)
Missing data	1.6 (9)	1.7 (11)	1.6 (7)	1.6 (7)
**Maternal education**				
≤ secondary school	29.8 (171)	29.2 (185)	31.7 (140)	31.7 (140)
Post-secondary school	32.8 (188)	33.4 (212)	32.6 (144)	32.6 (144)
University degree	35.9 (206)	35.7 (226)	34.2 (151)	34.2 (151)
Missing data	1.6 (9)	1.7 (11)	1.6 (7)	1.6 (7)
**Household income at age 5 years**				
< 4000 SGD	33.6 (193)	31.1 (197)	35.1 (155)	35.1 (155)
4000–7999 SGD	25.8 (148)	27.9 (177)	27.8 (123)	27.8 (123)
≥8000 SGD	24.2 (139)	22.6 (143)	22.6 (100)	22.6 (100)
Missing data	16.4 (94)	18.5 (117)	14.5 (64)	14.5 (64)
**Movement behaviours, min/day**				
Sleep	485.0 ± 56.9	498.6 ± 51.8	481.9 ± 58.4	500.0 ± 52.0
Inactivity	539.8 ± 79.4	536.6 ± 76.8	543.2 ± 82.0	538.3 ± 78.1
Light physical activity	344.3 ± 47.0	334.5 ± 52.0	343.8 ± 48.7	332.2 ± 52.3
Moderate physical activity	62.3 ± 19.2	61.3 ± 21.2	62.4 ± 19.8	60.4 ± 21.1
Vigorous physical activity	8.8 ± 4.8	8.9 ± 5.7	8.7 ± 4.9	9.1 ± 6.1

SD, standard deviation; BMI, body mass index; SGD, Singapore Doller

### Latent profiles at age 5.5- and 8- years

**Fig. 2:**
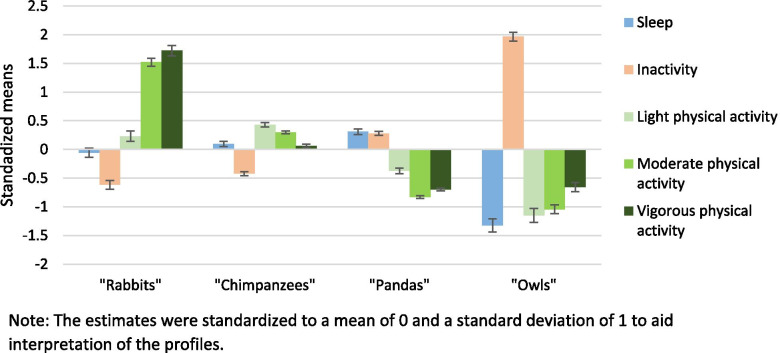
Description of 24-h movement behaviour profiles at age 5.5 and 8 years in children from the GUSTO cohort study

The four identified profile characteristics are illustrated in Fig. 2. The description of latent profiles in terms of estimated means, variance and proportion of participants in each profile are presented in Table 2. Compared to the overall sample, (i) children in the “Rabbits” profile had higher levels of PA, particularly MPA and VPA, lower inactivity and average night sleep duration, (ii) children in the “Chimpanzees” profile had higher levels of LPA and MPA, average VPA, lower inactivity and average night sleep duration, (iii) children in the “Pandas” profile had lower levels of PA, higher inactivity and longer night sleep, and (iv) children in the “Owls” profile had lower levels of PA, higher inactivity and very short night sleep.

**Table 2:** Descriptive statistics of latent profiles derived from 24-h movement behaviours in children aged 5.5 and 8 years in the GUSTO cohort study (n = 442)

	Profile 1	Profile 2	Profile 3	Profile 4
Profile name	“Rabbits” mean ± SD or % (n)	“Chimpanzees” mean ± SD or % (n)	“Pandas” mean ± SD or % (n)	“Owls” mean ± SD or % (n)
**24-h movement behaviours, min/day**				
Sleep	488.6 ± 51.3	497.3 ± 49.0	507.7 ± 46.0	424.0 ± 63.8
Inactivity	493.3 ± 67.3	507.0 ± 53.5	560.7 ± 47.8	687.9 ± 61.6
Light physical activity	350.1 ± 50.1	359.4 ± 39.0	321.6 ± 41.0	281.3 ± 58.2
Moderate physical activity	90.6 ± 16.8	67.1 ± 10.5	45.0 ± 8.9	41.4 ± 15.4
Vigorous physical activity	17.5 ± 6.0	9.3 ± 3.1	5.1 ± 2.1	5.5 ± 4.0
**Proportion of children assigned to the profile**				
5.5 years	12.4 (55)	51.1 (226)	24.4 (108)	12.0 (53)
8 years	16.5 (73)	39.1 (173)	36.7 (162)	7.7 (34)
**Proportion of children adhering to MVPA guideline**^**a**^				
5.5 years	100.0 (55)	93.8 (212)	12.0 (13)	24.5 (13)
8 years	100.0 (73)	94.2 (163)	13.0 (21)	20.6 (7)
**Proportion of children adhering to sleep guideline**^**b**^				
5.5 years	10.9 (6)	19.0 (43)	17.6 (19)	0.0 (0)
8 years	15.1 (11)	19.7 (34)	25.3 (41)	2.9 (1)

SD, standard deviation; MVPA, moderate-to-vigorous intensity physical activity

^a^ The proportion of children met MVPA (≥60 min/d) recommendation of WHO/Canadian guidelines

^b^ The proportion of children met (9–11 h/d) recommendation of Canadian guidelines

The majority of the children belonged to the profiles “Chimpanzees” (5.5 years: 51.1%; 8 years: 39.1%) and “Pandas” (5.5 years: 24.4%; 8 years: 36.7%); less than 20% belonged to the “Rabbits” and “Owls” profiles. The proportion of children in each profile meeting MVPA (≥60 min/d) and sleep (9–11 h/d) recommendations according to (WHO/Canadian) guidelines is presented in Table 2. Almost all children assigned to the “Rabbits” (100% at both time points) and the “Chimpanzees” profiles (5.5 years: 93.8%; 8 years: 94.2%) met the MVPA recommendations, while this proportion was small in the “Pandas” (5.5 years: 12.0%; 8 years: 13.0%) and the “Owls” profile (5.5 years: 24.5%; 8 years: 20.6%). The proportion of children meeting sleep recommendations was small across all profiles (≤25%), with almost none of the children in the “Owls” profile was meeting the sleep recommendation (Table 2).

### The transition of movement behaviour profile membership

**Fig. 3:**
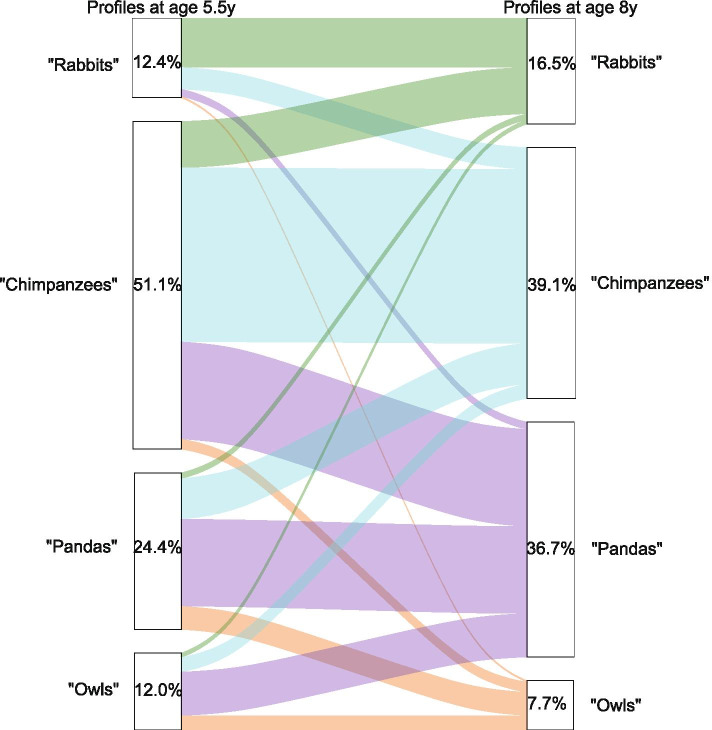
Transition of 24-h movement behaviour profiles from age 5.5 to 8 years in children from the GUSTO cohort study (*n* = 442)

The transition of 24-h movement behaviour profile membership from age 5.5 to 8 years is illustrated in Fig. 3. The latent transition probabilities of profile membership are presented in Supplementary Table 2. About half of the sample (49.3%) remained in the same profile and the other half changed profiles between age 5.5 and 8 years. Children in the “Rabbits” profile at age 5.5 years had the highest probability to remain in the same profile at 8 years (0.81), followed by children in the “Chimpanzees” (0.61) and “Pandas” profiles (0.59). The predominant patterns of change in the profiles were from “Rabbit” to “Chimpanzees” (0.12), “Chimpanzees” to “Pandas” (0.27), “Pandas” to “Owls” (0.19) or “Chimpanzees” (0.20) and “Owls” to “Pandas” (0.56).

### Associations of sex and ethnicity with profile membership and profile transition

Child sex was associated with profile membership at ages 5.5 and 8 years: compared to girls, boys were more likely to be in the “Rabbits” profile (adjusted OR [95% CI]: 3.6 [1.4, 9.7] and 4.5 [1.8, 10.9] at ages 5.5 and 8 years, respectively) and less likely to be in the “Pandas” profile (0.5 [0.3, 0.9] and 0.4 [0.2, 0.6]) at both ages. We found no associations between ethnicity and profile membership at ages 5.5 and 8 years (Table 3).

**Table 3:** 24-h movement behaviour profiles at age 5.5 and 8 years according to sex and ethnicity in children from the GUSTO cohort study (n = 442)

	Sex				Ethnicity					
	Girls % (n)	Boys % (n)	Boys vs girls Adjusted odds ratio (95% CI)^a^	Overall *p*-value	Chinese % (n)	Malay % (n)	Indian % (n)	Malay vs Chinese Adjusted odds ratio (95% CI)^a^	Indian vs Chinese Adjusted odds ratio (95% CI)^a^	Overall p-value
**Profile at 5.5 years**				< 0.001						0.669
“Rabbits”	5.7 (12)	18.5 (43)	3.6 (1.4, 9.7)		10.6 (27)	16.5 (19)	12.5 (9)	1.9 (0.8, 4.5)	1.2 (0.4, 3.5)	
“Chimpanzees”	47.6 (100)	54.3 (126)	1.0 (reference)		51.4 (131)	47.8 (55)	55.6 (40)	1.0 (reference)	1.0 (reference)	
“Pandas”	31.4 (66)	18.1 (42)	0.5 (0.3, 0.9)		27.1 (69)	16.5 (19)	27.8 (20)	0.6 (0.3, 1.4)	0.9 (0.4, 2.0)	
“Owls”	15.2 (32)	9.1 (21)	0.5 (0.3, 1.0)		11.0 (28)	19.1 (22)	4.2 (3)	2.1 (1.0, 4.4)	0.3 (0.0, 1.5)	
**Profile at 8 years**				< 0.001						0.461
“Rabbits”	5.7 (12)	26.3 (61)	4.5 (1.8, 10.9)		13.7 (35)	21.7 (25)	18.1 (13)	2.3 (1.1, 5.2)	1.3 (0.5, 3.3)	
“Chimpanzees”	34.8 (73)	43.1 (100)	1.0 (reference)		40.8 (104)	31.3 (36)	45.8 (33)	1.0 (reference)	1.0 (reference)	
“Pandas”	49.5 (104)	25.0 (58)	0.4 (0.2, 0.6)		38.4 (98)	36.5 (42)	30.6 (22)	1.4 (0.7, 2.7)	0.6 (0.3, 1.4)	
“Owls”	10.0 (21)	5.6 (13)	0.4 (0.2, 1.0)		7.1 (18)	10.4 (12)	5.6 (4)	2.3 (0.9, 5.9)	0.6 (0.2, 2.4)	

CI, confidence interval

^a^Sex and ethnicity were mutually adjusted for each other in the models

Odds ratios and 95% CIs were determined by multinomial logistic regression using the Bolck-Croon-Hagenarrs (BCH) method

The sex- and ethnicity-specific transition probabilities of profile membership is shown in Supplementary Table 2. Compared to girls, the probability of boys to remain in the “Rabbits” profile was higher (0.91 vs 0.36) and the probability to remain in the “Pandas” profile was lower (0.37 vs 0.72). The probability of moving from the “Chimpanzees” to the “Pandas” profile was lower in boys than girls (0.20 vs 0.35). It appears that the transition of profile membership was similar across the ethnicity of children.

Additionally, Supplementary Table 3 shows the 24 h movement behaviour profiles at age 5.5 and 8 years according to characteristics of children and their family: the proportion of children assigned in each profile varied by maternal education, but not by children’s BMI, maternal age and household income. Children of mothers with higher educational level were less likely to be assigned to the “Owls” profile at both time points, compared to children of mothers with lower educational level (5.5 years: 15.4% vs 44.2%; 8 years: 8.8% vs 58.8%).

### The sensitivity analysis of latent profile analysis

Latent profile analyses were repeated in the maximum samples at age 5.5 (*n* = 574) and 8 years (*n* = 634), and yielded similar profiles (Supplementary Table 4) and result for the associations of sex and ethnicity with profile membership at each time point.

## Discussion

This study described accelerometer-measured 24-h movement behaviour latent profiles at age 5.5 and 8 years in a multi-ethnic Asian population. The evidence from this study suggests that, based on their time use in LPA, MPA, VPA, inactivity and sleep at both ages, children can be classified into four distinct profiles: the profile with higher PA, lower inactivity and average night sleep duration (“Chimpanzees”) was most prevalent, followed by the profile with lower PA, higher inactivity and longer night sleep duration (“Pandas”). However, the two more extreme profiles, comprising children with very high VPA/MPA, low inactivity levels and average night sleep duration on the one hand (“Rabbits”), and low PA, high inactivity, and low night sleep duration on the other hand (“Owls”) represent sizable and from a health promotion perspective potentially important populations. In addition, this study provides novel evidence on the transition between profiles with increasing age: while about 50% of children stayed in their profile, the predominant transition among the remaining children occurred towards lower PA, higher inactivity and longer night-sleep patterns. Differences between boys and girls in terms of their profile membership but also their transition with age were apparent.

Our results generally support the most recent systematic review on lifestyle behaviour patterns among children aged 5–12 years: the review found seven studies that investigated clustering of children based on PA and SB patterns and identified healthy (high PA and low SB), unhealthy (low PA and high SB) and the mixture of healthy and unhealthy behaviours (both PA and SB were high or low) [10]. Few studies included sleep and all these studies also included dietary factors to determine the clusters/profiles [10, 12, 13], and none of the studies reported the clusters/profiles exclusively based on movement behaviours across a 24-h day. In the present study, we could not classify the profiles as healthy or unhealthy, because the health effects of single movement behaviours may be counteracting each other and the health outcomes associated with these profiles are currently unclear. However, “Rabbits” and “Chimpanzees” profiles can be considered relatively healthy profiles as they had a higher PA, lower inactivity and average sleep duration with almost all children meeting the recommended 60 min of MVPA. However, the proportions of children meeting the sleep guideline (9–11 h) in our study were very low across all profiles. While the “Pandas” profile (lower PA, higher inactivity and longer sleep duration) has unhealthy PA and inactivity patterns, this profile may still have some health-promoting attributes due to its longer sleep duration. Therefore, the “Pandas” profile may be considered as a combination of healthy sleep and unhealthy PA and inactivity patterns, while the “Owls” profile (lower PA, higher inactivity and shorter sleep duration) is characterised by a generally unhealthy movement behaviour pattern. Further investigation of the health outcomes associated with these profiles may help determine to what extent movement behaviour profiles are healthy or unhealthy.

This is the first study reporting movement profiles in children based on the full continuum of 24-h movement behaviours, including LPA, MPA, VPA, inactivity and sleep. This approach aligns with the Framework for Viable integrative Research in Time-Use Epidemiology (VIRTUE) that emphasizes the importance of using an integrated approach to studying time-use balance in movement behaviours and their prevalence in populations [11, 53]. Previous studies among children (aged 5–12 years) did not take sleep into consideration, nor did they consider MPA and VPA separately [20, 54]. This, however, seems important, since we identified the “Rabbits” profile that had a high level of VPA, and this might have important health implications: evidence suggests that greater amounts of VPA are associated with favourable cardio-metabolic health outcomes and improved cardiorespiratory fitness [55, 56]. WHO strongly recommends at least three days of vigorous-intensity activities per week for children and adolescents aged 5–17 years in the 2020 guidelines [38]. Similar to our approach, Gupta et al. identified four 24-h movement behaviour profiles. The authors also labelled the profiles with animal names. However, comparison of profile characteristics in both studies remains challenging given that their study was conducted among adults and focussed on occupational and leisure-time LPA, MVPA, SB and standing and bedtime [53].

The vast majority of previous studies among children were cross-sectional, and only one investigated the profiles of movement behaviours longitudinally [20]. Jago et al. investigated PA and SB profiles, but not sleep, among UK children in the B-PROACTIVE study. Their result suggested greater movement between profiles. While about 30% of children were in the same profile at age 6 and 9 years, the majority moved towards lower MVPA and higher SB at the three-year follow-up [20]. Transition patterns in the present study may not be directly comparable with these findings from the UK, because sleeping behaviour was not included in the UK study. Our results showed that nearly half of the children were in the same profile at age 5.5 and 8 years, which confirms evidence on the tracking nature of movement behaviours with increasing age [7–9]. Considering only PA and inactivity, we also found that a substantial proportion of children moved to the profiles with lower PA and higher inactivity pattern, resulting in a greater proportion of children in the “Pandas” profile at age 8 years (5.5 years: 24%; 8 years: 37%). However, moving to the “Pandas” profile may also be considered as a positive evolution in sleep pattern as children in this profile demonstrate a longer sleep duration. Similarly, a meaningful proportion of children moved to “Rabbits” profile, depicting more favourable movement behaviours. Hence, our findings demonstrate that positive evolutions exist in our study population among Asian children, which is encouraging for future health promotion activities and warrants further investigation to better understand these transitions between profiles.

The potential mechanism of changing profile memberships between age 5.5 to 8 years is currently not well understood since this is the first study to describe such transitions. Evidence suggests that multiple factors, including personal, parental/family, social and environmental factors, could explain the changes in movement behaviours of children as they transit between schools or with increasing school years [17, 57–59]. For instance, changing from kindergarten to primary school (grade 2) between ages 5.5 to 8 years may require the children to sit longer for academic activities and increase the use of screen devices [17, 58]. Moreover, napping is common at kindergarten but not at primary school in Singapore, which might explain the predominant changes of moving towards profiles with lower PA and higher inactivity and/or longer night sleep duration at age 8 years. Similarly, children might have been motivated differently by schools/parents/pear groups to practice healthier behaviour such as participating in PA or extracurricular organised sports and maintain sleep hygiene [60–62], which could partly explain why some children moved towards profiles with higher PA and lower inactivity or higher night sleep duration.

The present study also found that sex was associated with profile membership in our population: as compared to girls, boys were approximately four times more likely to be in the “Rabbits” profile. These results generally support the findings of previous systematic reviews which reported that a greater proportion of boys were assigned to the clusters with higher PA, whereas a greater proportion of girls was assigned in clusters with lower PA [10, 29]. We also explored the transition of profile membership from age 5.5 to 8 years and found that girls had a higher probability of moving from the more active “Chimpanzees” profile to the less active “Pandas” profile than boys. These findings are generally consistent with findings of the previous prospective study from the UK, which reported that girls were more likely to move into less active profiles from age 6 to 9 years [20]. A recent systematic review also reported that girls had a higher reduction in PA time compared to boys between the ages of 4 and 9 [63]. This warrants further investigation to examine the mechanisms underlying different patterns of profile membership and transition in profile membership, which will ultimately help in the development of more targeted health promotion strategies. Some possible associations between ethnicity and profile memberships were noted, but they were not consistent. This may also be due to a lack of statistical power, and requires further investigation with larger sample size to understand ethnic differences in movement behaviour patterns of children in Asia.

A major strength of the study is the use of accelerometers with non-removable wrist strap, allowing us to collect high-quality data across the full continuum of 24-h movement behaviours seamlessly. The longitudinal study design and repeated measures helped to examine changes in profile membership during the transition from pre-school to school age. Some limitations have to be acknowledged, though. Our study is not representative of the general Singaporean population, and the proportion of children who completed both time points was only about 40% of the original study population, which further reduces generalizability and statistical power. We used wrist-worn accelerometers to measure movement behaviour which was associated with high compliance. However, unlike hip- or thigh-worn accelerometers that are closer to the centre of the body, wrist-worn accelerometer have a lower correspondence with whole body movements [14]. For instance, wrist-worn accelerometers could register acceleration while sitting [64] and are not able to differentiate SB from other types of inactivity, including nap time [14, 16, 31, 32]. Consequently, it was not clear whether lower night sleep at younger age was due to differences in napping behaviour in some children, particularly among children assigned in the “Owls” profile, and this warrants further investigation. Moreover, movement behaviour patterns were measured only at two time points, thus we could not measure the longer-term trends in movement behaviours among children; further follow-up of our cohort will shed light on this. Nonetheless, our findings are important by taking a contemporary perspective to identify distinct patterns across the full continuum of movement behaviours in a full day. This information can be useful in highlighting opportunities for research and designing strategies to improve movement behaviours for health.

## Conclusions

Our study among a multi-ethnic population of children in Singapore identified four distinct movement behaviour profiles: “Rabbits (higher PA, particularly MPA and VPA, lower inactivity and average night sleep), “Chimpanzees” (higher PA, lower inactivity and average night sleep), “Pandas” (lower PA, higher inactivity and longer night sleep) and “Owls” (lower levels of PA, higher inactivity and very short night sleep duration). “Chimpanzees” followed by “Pandas” profile were the most prevalent. These findings demonstrate the importance of considering the full continuum of movement behaviours as compared to investigating them separately. With increasing age, almost half of the sample remained in their profile, highlighting the importance of engagement in healthy movement behaviours early in life. Among those who changed profile, a ‘downward’ trend towards less active movement behaviours was most common (especially “Chimpanzees” to “Pandas” profile). Compared to girls, boys were more likely to be in the very active “Rabbits” profile and less likely to be in the more inactive “Pandas” profile at both ages. This study provides novel evidence on classifying children based on the full spectrum of 24 h movement behaviour patterns, and this is the vital step to identify healthier movement behaviour patterns and their determinants. Therefore, further research is warranted to confirm our findings and to understand health outcomes and the determinants of unhealthy movement behaviour profiles, as well as transitions between profiles. Subsequently, this evidence will contribute to the development of more targeted and potentially more effective intervention strategies to promote healthier movement behaviours among children.

## Supplementary Information

**Additional file 1.**

**Additional file 2.**

**Additional file 3.**

**Additional file 4.**

## Data Availability

The dataset supporting the conclusions of this article can be made available upon request and after approval by the GUSTO Executive Committee.
