# Supplementary material for: 24-hour movement behaviour profiles and their transition in children aged 5.5 and 8 years – findings from a prospective cohort study
Source: Int J Behav Nutr Phys Act. 2021 Nov 6;18:145. doi: 10.1186/s12966-021-01210-y (PMC8572484; doi:10.1186/s12966-021-01210-y)
Supplement: Supplementary file 1 — Additional file 1. [file 12966_2021_1210_MOESM1_ESM.docx]

Supplementary Table 1: Model fit indices and indicators of latent profile analysis models

| Profiles | LL | AIC^1^ | BIC^1^ | SSBIC^1^ | LMR^2^ | BLRT^2^ | Smallest class size  (in % of the total sample) | Relative entropy |
| --- | --- | --- | --- | --- | --- | --- | --- | --- |
| **5.5 years** |  |  |  |  |  |  |  |  |
| 2 | -10437.744 | 20907.487 | 20972.948 | 20922.172 | 0.0020 | <0.001 | 30.7 | 0.754 |
| 3 | -10357.010 | 20758.019 | 20848.028 | 20778.210 | 0.2787 | <0.001 | 11.7 | 0.794 |
| 4 | -10297.113 | 20650.226 | 20764.782 | 20675.923 | 0.3188 | <0.001 | 5.7 | 0.807 |
| 5 | -10239.214 | 20546.428 | 20685.532 | 20577.632 | 0.1568 | <0.001 | 5.4 | 0.810 |
| 6 | -10200.330 | 20480.660 | 20644.313 | 20517.371 | 0.7035 | <0.001 | 4.7 | 0.807 |
| 7 | -10155.248 | 20402.497 | 20590.697 | 20444.714 | 0.1927 | <0.001 | 2.0 | 0.809 |
| **8 years** |  |  |  |  |  |  |  |  |
| 2 | -10526.302 | 21084.604 | 21150.065 | 21099.289 | 0.0053 | <0.001 | 19.6 | 0.860 |
| 3 | -10404.011 | 20852.022 | 20942.031 | 20872.213 | 0.0499 | <0.001 | 14.2 | 0.792 |
| 4 | -10338.649 | 20733.298 | 20847.855 | 20758.996 | 0.0862 | <0.001 | 5.8 | 0.802 |
| 5 | -10299.673 | 20667.347 | 20806.451 | 20698.551 | 0.8162 | <0.001 | 6.3 | 0.813 |
| 6 | -10265.894 | 20611.787 | 20775.440 | 20648.498 | 0.1647 | <0.001 | 5.0 | 0.799 |
| 7 | -10233.723 | 20559.447 | 20747.647 | 20601.664 | 0.4476 | <0.001 | 0.5 | 0.808 |
| Configural similarity for 4-profile model | -20635.762 | 41383.524 | 41612.637 | 41434.919 | - | - | - | 0.804 |
| Structural similarity (means) for 4-profile model | -20679.634 | 41431.268 | 41464.307 | 41464.307 | - | - | - | 0.796 |
| Dispersion similarity (means and variances) for 4-profile model | -20574.352 | 41240.704 | 41428.905 | 41282.922 | - | - | - | 0.776 |

LL, Loglikelihood; AIC, Akaike information criterion; BIC, Bayesian information criterion; SSBIC, sample size-adjusted Bayesian information criterion; LMR, Lo-Mendell-Rubin likelihood ratio test; BLRT, Bootstrapped likelihood ratio test

^1^ lower AIC, BIC, SSBIC and LL indicate better model fit

^2^ p-value for test comparing the current number of classes to a model with one fewer class
