# Supplementary material for: 24-hour movement behaviour profiles and their transition in children aged 5.5 and 8 years – findings from a prospective cohort study
Source: Int J Behav Nutr Phys Act. 2021 Nov 6;18:145. doi: 10.1186/s12966-021-01210-y (PMC8572484; doi:10.1186/s12966-021-01210-y)
Supplement: Supplementary file 2 — Additional file 2. [file 12966_2021_1210_MOESM2_ESM.docx]

Supplementary Table 2: Sex and ethnic-specific probabilities of changes in profile membership from age 5.5 to 8 years in children from the GUSTO cohort study (n=442)

|  | **Profiles at age 8 years** | | | |
| --- | --- | --- | --- | --- |
|  | “Rabbits” | “Chimpanzees” | “Pandas” | “Owls” |
| **Profiles at age 5.5 years** |  |  |  |  |
| All children |  |  |  |  |
| “Rabbits” | 0.81 | 0.12 | 0.05 | 0.02 |
| “Chimpanzees” | 0.10 | 0.61 | 0.27 | 0.02 |
| “Pandas” | 0.02 | 0.20 | 0.59 | 0.19 |
| “Owls” | 0.06 | 0.14 | 0.56 | 0.24 |
| Boys |  |  |  |  |
| “Rabbits” | 0.91 | 0.07 | 0.02 | 0.00 |
| “Chimpanzees” | 0.13 | 0.63 | 0.20 | 0.04 |
| “Pandas” | 0.05 | 0.43 | 0.37 | 0.16 |
| “Owls” | 0.17 | 0.05 | 0.61 | 0.18 |
| Girls |  |  |  |  |
| “Rabbits” | 0.36 | 0.34 | 0.18 | 0.13 |
| “Chimpanzees” | 0.07 | 0.57 | 0.35 | 0.00 |
| “Pandas” | 0.00 | 0.07 | 0.72 | 0.21 |
| “Owls” | 0.00 | 0.19 | 0.54 | 0.27 |
| Chinese |  |  |  |  |
| “Rabbits” | 0.83 | 0.17 | 0.00 | 0.00 |
| “Chimpanzees” | 0.08 | 0.63 | 0.29 | 0.01 |
| “Pandas” | 0.02 | 0.25 | 0.57 | 0.17 |
| “Owls” | 0.03 | 0.03 | 0.66 | 0.28 |
| Malay |  |  |  |  |
| “Rabbits” | 0.72 | 0.08 | 0.14 | 0.07 |
| “Chimpanzees” | 0.17 | 0.49 | 0.30 | 0.05 |
| “Pandas” | 0.02 | 0.00 | 0.59 | 0.40 |
| “Owls” | 0.10 | 0.24 | 0.50 | 0.16 |
| Indian |  |  |  |  |
| “Rabbits” | 0.88 | 0.02 | 0.10 | 0.00 |
| “Chimpanzees” | 0.11 | 0.70 | 0.18 | 0.02 |
| “Pandas” | 0.04 | 0.24 | 0.60 | 0.12 |
| “Owls” | 0.00 | 0.33 | 0.14 | 0.53 |
|  |  |  |  |  |
| Values are derived from latent transition analyses | | | | |
