# Supplementary material for: 24-hour movement behaviour profiles and their transition in children aged 5.5 and 8 years – findings from a prospective cohort study
Source: Int J Behav Nutr Phys Act. 2021 Nov 6;18:145. doi: 10.1186/s12966-021-01210-y (PMC8572484; doi:10.1186/s12966-021-01210-y)
Supplement: Supplementary file 3 — Additional file 3. [file 12966_2021_1210_MOESM3_ESM.docx]

Supplementary Table 3: 24h movement behaviour profiles at age 5.5 and 8 years according to characteristics of children and their family in the GUSTO cohort study

|  | **24h movement behaviour profiles at age 5.5 years** | | | | **p-value** | **24h movement behaviour profiles at age 8 years** | | | | **p-value** |
| --- | --- | --- | --- | --- | --- | --- | --- | --- | --- | --- |
|  | “Rabbits”  % (n) or  mean ± SD | “Chimpanzees”  % (n) or  mean ± SD | “Pandas”  % (n) or  mean ± SD | “Owls”  % (n) or  mean ± SD |  | “Rabbits”  % (n) or  mean ± SD | “Chimpanzees”  % (n) or  mean ± SD | “Pandas”  % (n) or  mean ± SD | “Owls”  % (n) or  mean ± SD |  |
| **BMI at age 5 years** |  |  |  |  | 0.626 |  |  |  |  | 0.194 |
| Below median (<15.0 kg/m^2^) | 52.9 (27) | 46.1 (101) | 51.9 (54) | 44.2 (23) |  | 45.7 (32) | 54.2 (90) | 44.9 (70) | 38.2 (13) |  |
| Median and above (≥15.0 kg/m^2^) | 47.1 (24) | 53.9 (118) | 48.1 (50) | 55.8 (29) |  | 54.3 (38) | 45.8 (76) | 55.1 (86) | 61.8 (21) |  |
| **Maternal age at recruitment** |  |  |  |  | 0.290 |  |  |  |  | 0.403 |
| <27 years | 16.7 (9) | 26.0 (58) | 16.0 (17) | 23.1 (12) |  | 25.0 (18) | 24.6 (42) | 17.7 (28) | 23.5 (8) |  |
| 27-33 years | 44.4 (24) | 39.9 (89) | 45.2 (48) | 34.6 (18) |  | 43.1 (31) | 40.4 (69) | 44.3 (70) | 26.5 (9) |  |
| >33 years | 38.9 (21) | 34.1 (76) | 38.7 (41) | 42.3 (22) |  | 31.9 (23) | 35.1 (60) | 38.0 (60) | 50.0 (17) |  |
| **Maternal education** |  |  |  |  | 0.007* |  |  |  |  | <0.001* |
| ≤ secondary school | 31.5 (17) | 32.3 (72) | 26.4 (28) | 44.2 (23) |  | 30.6 (22) | 27.5 (47) | 32.3 (51) | 58.8 (20) |  |
| Post-secondary school | 25.9 (14) | 32.3 (72) | 34.9 (37) | 40.4 (21) |  | 25.0 (18) | 32.2 (55) | 38.0 (60) | 32.4 (11) |  |
| University degree | 42.6 (23) | 35.4 (79) | 38.7 (41) | 15.4 (8) |  | 44.4 (32) | 40.4 (69) | 29.8 (47) | 8.8 (3) |  |
| **Household income at age 5 years** |  |  |  |  | 0.206 |  |  |  |  | 0.106 |
| <4000 SGD | 41.9 (18) | 41.2 (79) | 35.4 (34) | 51.1 (24) |  | 39.7 (25) | 40.4 (59) | 38.4 (53) | 58.1 (18) |  |
| 4000-7999 SGD | 34.9 (15) | 31.3 (60) | 34.4 (33) | 31.9 (15) |  | 33.3 (21) | 30.1 (44) | 35.5 (49) | 29.0 (9) |  |
| ≥8000 SGD | 23.3 (10) | 27.6 (53) | 30.2 (29) | 17.0 (8) |  | 27.0 (17) | 29.5 (43) | 26.1 (36) | 12.9 (4) |  |
| SD, standard deviation; BMI, body mass index; SGD, Singapore Dollar  *Significant p-value; p-values are based on Chi-square test using BCH method | | | | |  |  |  |  |  |  |
