# Supplementary material for: 24-hour movement behaviour profiles and their transition in children aged 5.5 and 8 years – findings from a prospective cohort study
Source: Int J Behav Nutr Phys Act. 2021 Nov 6;18:145. doi: 10.1186/s12966-021-01210-y (PMC8572484; doi:10.1186/s12966-021-01210-y)
Supplement: Supplementary file 4 — Additional file 4. [file 12966_2021_1210_MOESM4_ESM.docx]

Supplementary Table 4: Descriptive statistics of latent profiles derived from 24-h movement behaviours in children aged 5.5 (n=574) and 8 (n=634) years in the GUSTO cohort study for maximum sample

|  | **Profile 1** | **Profile 2** | **Profile 3** | **Profile 4** |
| --- | --- | --- | --- | --- |
| Profile name | “Rabbits”  mean ± SD  or  % (n) | “Chimpanzees”  mean ± SD  or  % (n) | “Pandas”  mean ± SD  or  % (n) | “Owls”  mean ± SD  or  % (n) |
| **Proportion of children assigned to the profile** |  |  |  |  |
| 5.5 years | 11.5 (66) | 53.3 (306) | 24.4 (140) | 11.0 (62) |
| 8 years | 16.6 (105) | 40.1 (254) | 35.3 (224) | 8.0 (51) |
| **Proportion of children adhering to MVPA guideline^a^** |  |  |  |  |
| 5.5 years | 100.0 (66) | 93.8 (287) | 12.9 (18) | 24.2 (15) |
| 8 years | 100.0 (105) | 95.3 (242) | 14.7 (33) | 19.6 (10 ) |
| **Proportion of children adhering to sleep guideline^b^** |  |  |  |  |
| 5.5 years | 16.7 (11) | 18.6 (57) | 16.4 (23) | 0.0 (0) |
| 8 years | 12.4 (13) | 20.5 (52) | 22.3 (50) | 5.9 (3) |
| SD, standard deviation; MVPA, moderate-to-vigorous intensity physical activity  ^a^ The proportion of children met MVPA (≥60 min/d) recommendation of WHO/Canadian guidelines  ^b^ The proportion of children met (9-11 h/d) recommendation of Canadian guidelines | | | | |
